# A comprehensive first-trimester predictive model for preeclampsia based on multi-indicators and machine learning: A retrospective single-center study

**DOI:** 10.1097/MD.0000000000045555

**Published:** 2025-11-21

**Authors:** Haixia Liang, Xuejing Zhao, Ying Zhang, Yujie Wu, Han Wu, Zehui Zhang, Ying He

**Affiliations:** a Department of Obstetrics and Gynecology, Xijing Hospital the 986th Hospital Department, The Fourth Military Medical University, Xi’an, Shaanxi, China.

**Keywords:** early pregnancy, inflammation, machine learning, placental growth factor, predictive model, preeclampsia, uterine artery pulsatility index

## Abstract

Preeclampsia (PE) is a severe, pregnancy-specific disorder that significantly contributes to maternal and perinatal morbidity and mortality. Its unpredictable onset after 20 weeks of gestation underscores the critical need for effective early prediction and intervention. This study aimed to develop a comprehensive predictive model for PE using a wide array of maternal, biophysical, biochemical, and hematological indicators from the 1st trimester. This retrospective study included 100 pregnant individuals with singleton gestations (50 PE, 50 controls). Various early pregnancy indicators, including hematological, biochemical, inflammatory, angiogenic, and biophysical markers, were collected. Least absolute shrinkage and selection operator regression was used for feature selection. Subsequently, 7 different machine learning (ML) algorithms were employed for model development. Model performance was evaluated using receiver operating characteristic curves. An independent external validation cohort of 70 participants (35 PE, 35 controls) was used to confirm the model’s generalizability. Baseline characteristics showed significantly higher early pregnancy systolic blood pressure and diastolic blood pressure in the PE group (*P* < .001). Early pregnancy indicator comparisons revealed the PE group had significantly higher median white blood cell count, neutrophil count, monocyte count, and C-reactive protein (CRP) levels, and lower median hemoglobin and hematocrit. Derived indices like the neutrophil-to-lymphocyte ratio (NLR) were significantly higher (*P* < .001). Crucially, placental growth factor (PlGF) levels were significantly lower (*P* < .001), while uterine artery pulsatility index (UtAPI) was significantly higher (*P* < .001). Least absolute shrinkage and selection operator regression identified 12 key predictive features, including PlGF, UtAPI, CRP, and NLR. Among the ML models, the neural network model demonstrated the highest predictive performance, with an area under the curve of 0.917. The model maintained strong performance (area under the curve = 0.838) in external validation. SHapley Additive exPlanations analysis confirmed PlGF, UtAPI, CRP, and NLR as the most influential features. We developed a robust predictive model for PE based on early pregnancy biomarkers and ML techniques. The neural network model demonstrated superior discriminative ability in both internal and external validation cohorts. Early identification of high-risk pregnancies using this model could facilitate timely interventions, such as low-dose aspirin, potentially improving maternal and fetal outcomes. Further multi-center prospective studies are warranted to validate the model on a broader scale.

## 1. Introduction

Preeclampsia (PE) is a severe, pregnancy-specific disorder characterized by new-onset hypertension and proteinuria after 20 weeks of gestation, impacting 2% to 8% of pregnancies globally.^[1,2]^ It stands as a leading cause of maternal and perinatal morbidity and mortality, significantly contributing to premature birth, fetal growth restriction, placental abruption, and long-term cardiovascular complications for both mother and child.^[3,4]^ The unpredictable nature of PE’s onset and progression highlights the urgent need for effective early prediction and intervention strategies to mitigate its devastating consequences.

Traditional risk factor screening for PE, often relying on maternal history and demographic characteristics, has demonstrated limited predictive accuracy.^[5,6]^ This necessitates the development of more robust and comprehensive predictive models, especially in early pregnancy. Identifying high-risk women during the 1st trimester is crucial, as this allows for timely implementation of preventative measures, such as low-dose aspirin, which are most effective when administered early, potentially altering the disease trajectory and improving pregnancy outcomes.^[7]^

The pathogenesis of PE is intricate and multifactorial, involving abnormal placental development, maternal systemic inflammation, endothelial dysfunction, and impaired vascular remodeling.^[8–11]^ These pathological processes often commence in the 1st trimester, long before clinical symptoms emerge. Consequently, various circulating biomarkers, biophysical markers, and maternal characteristics reflecting these underlying dysfunctions have surfaced as promising candidates for early PE prediction. Recent advancements emphasize combining multiple markers to enhance predictive performance. For instance, integrating maternal demographic data with biochemical markers like placental growth factor (PlGF) and biophysical markers such as uterine artery pulsatility index (UtAPI) has been shown to significantly improve the detection rate for early-onset PE.^[12,13]^ Furthermore, emerging evidence suggests the involvement of systemic inflammatory responses and metabolic disturbances in the early stages of PE, with routine complete blood count parameters and derived ratios like neutrophil-to-lymphocyte ratio (NLR) and platelet-to-lymphocyte ratio (PLR) offering insights into systemic inflammation.^[14–16]^ However, the complex interplay between these diverse factors necessitates a more integrated approach to fully leverage their predictive potential.

This study developed and validated a comprehensive predictive model for PE during the 1st trimester (up to 12 weeks of gestation) by integrating a broad spectrum of maternal demographic, historical, biophysical, biochemical, hematological, inflammatory, and coagulation parameters. By employing advanced machine learning (ML) techniques, including least absolute shrinkage and selection operator (LASSO) regression for rigorous feature selection and subsequent model construction using multiple algorithms (culminating in the superior performance of the neural network [NNET] model), we aimed to establish a robust early prediction system. This system is intended to facilitate the early identification of high-risk pregnancies, enabling timely interventions and ultimately improving maternal and fetal outcomes.

## 2. Methods

### 2.1. Study design and participants

This retrospective study was conducted at Xijing Hospital the 986th Hospital Department, The Fourth Military Medical University. We included 100 pregnant individuals with singleton gestations for model development and internal testing. The *PE group* consisted of patients diagnosed with PE according to the “Guidelines for the Diagnosis and Treatment of Hypertensive Disorders in Pregnancy (2020)” by the Chinese Medical Association, Obstetrics and Gynecology Branch, who delivered between January 1, 2024, and January 31, 2025.

The criteria used for diagnosis are as follows: systolic blood pressure (SBP) ≥ 140 mm Hg and/or diastolic blood pressure (DBP) ≥ 90 mm Hg, with either a urine protein quantification of ≥ 0.3 g/24 h, a urine protein/creatinine ratio of ≥ 0.3, or a random urine protein result of ≥ (1+). In the absence of proteinuria, new-onset hypertension with any of the following organ or system involvements: heart, lungs, liver, kidneys, or abnormalities in the blood, digestive, or nervous systems, or placental-fetal complications.

The *control group* comprised low-risk pregnant individuals with singleton gestations, no chronic diseases, or medication use, who delivered within the same timeframe. Exclusion criteria for both groups included preexisting hypertension, gestational hypertension, acute or chronic inflammatory conditions at the time of data collection, and miscarriage before 28 weeks of gestation. The control group comprised a cohort of low-risk pregnant individuals with singleton gestations who delivered within the same timeframe and were selected in a 1:1 ratio to match the PE group. This balanced design is a widely accepted approach in ML for rare events, as it ensures that the model has a sufficient number of positive cases to learn from, thereby improving predictive performance and mitigating issues related to class imbalance.

Patient information was retrieved from the hospital’s electronic database. For external validation, an independent cohort of 70 pregnant individuals with singleton gestations (35 in PE group, 35 in control group) was additionally recruited from February 1, 2025, to May 31, 2025. The equal 1:1 ratio for this cohort was maintained to ensure a rigorous and unbiased external validation of the model’s generalizability. This study received ethical approval from the Ethics Committee of Xijing Hospital the 986th Hospital Department, The Fourth Military Medical University.

### 2.2. Data collection and measurements

A comprehensive set of early pregnancy (prior to 12 weeks of gestation) theoretical indicators were collected from all participants. White blood cell (WBC) related parameters, including WBC count, neutrophil count, lymphocyte count, monocyte count, eosinophil count, basophil count, and C-reactive protein (CRP), were measured using the Mindray D7-CRP blood cell analyzer. This analyzer was also used to obtain red blood cell (RBC) related parameters: RBC count, hemoglobin (Hb), hematocrit, mean corpuscular volume, mean corpuscular hemoglobin, mean corpuscular hemoglobin concentration, red cell distribution width-coefficient of variation (RDW-CV), and red cell distribution width-standard deviation.

Derived calculated indices were also determined, specifically the NLR, monocyte-to-lymphocyte ratio (MLR), PLR, and platelet count to mean platelet volume ratio. The formulas used for these calculations were:


$$\begin{array}{l} \begin{array}{l} NLR\;=\;Neutrophil\;count/Lymphocyte\;count;\;MLR\;=\;Monocyte\;count/Lymphocyte\;count;\;PLR\;=\;Platelet\;count/Lymphocyte\;count;\;PC/MPV\;=\;Platelet\;count/Mean\;Platelet\;Volume.\; \end{array} \end{array}$$

Angiogenic factors included PlGF, which was measured using the Hebei Tewent T-meter1. Biophysical markers involved UtAPI, measured with the Samsung HearW10 ultrasound system.

Clinical parameters such as blood pressure, age, and body mass index were collected. Maternal history details, including PE history or family history (1st-degree relatives), chronic diseases (hypertension, diabetes, kidney disease, autoimmune diseases), primiparity, and multiple gestation, were also documented.

Liver function indicators (alanine aminotransferase, aspartate aminotransferase [AST], lactate dehydrogenase, and serum albumin) and kidney function indicators (serum creatinine [Cr], blood urea nitrogen, and uric acid [UA]) were measured using the Roche 702 automatic biochemical analyzer. Coagulation parameters, specifically fibrinogen and d-dimer (d-Dimer), were assessed using the Sysmex CS-2000i coagulation analyzer.

Information regarding the occurrence of PE was meticulously documented throughout the study as the primary outcome of interest.

### 2.3. Statistical analysis

Statistical analyses were performed using R software (V4.4.1; https://www.r-project.org/). The normality of continuous variables were assessed using the Shapiro–Wilk test. Continuous variables not conforming to a normal distribution were presented as median and interquartile range (IQR) and compared using the Mann–Whitney *U* test. Normally distributed continuous variables were presented as mean ± standard deviation and compared using *t*-tests. Categorical variables were expressed as numbers and percentages and compared using the chi-square test or Fisher exact probability test.

To identify the most relevant predictors from the extensive set of collected variables, LASSO regression was utilized for feature selection. We chose LASSO because it is a robust regularization method well-suited for high-dimensional data. It performs automatic feature selection by shrinking the coefficients of less important variables to zero, thus creating a parsimonious and highly interpretable model while effectively mitigating the risk of overfitting.

The variables identified by LASSO were then used to construct a robust predictive model using 7 different ML algorithms: random forest (RF), support vector machine (SVM), generalized linear model (GLM), gradient boosting machine (GBM), K-nearest neighbors (KNN), NNET, and decision tree (DT). To address the issue of overfitting, each model was trained and optimized using *k*-fold repeated cross-validation (5-fold) on the training dataset. This process allowed us to fine-tune hyperparameters and ensure the model’s robust performance on unseen data.

To enhance model interpretability, SHapley Additive exPlanations (SHAP) values were calculated to provide insights into variable importance and the contribution of individual features. The clinical utility of the developed model will be rigorously assessed through several methods: a Nomogram will be constructed to create a risk scoring system; calibration curves will be generated to evaluate the consistency between predicted probabilities and observed outcomes; and decision curve analysis will be performed to quantify the net benefit of using the model in clinical decision-making across various risk thresholds. Furthermore, multivariable logistic regression analysis will be conducted to examine associations between selected predictors and PE, and receiver operating characteristic (ROC) curve analysis will be performed for individual indicators and combinations of indicators, with optimal cutoff values determined by maximizing the Youden Index. Statistical significance will be defined as *P* < .05.

Finally, we have carefully consulted the Transparent Reporting of a Multivariable Prediction Model for Individual Diagnosis and Prognosis and Prediction model Risk of Bias ASsessment Tool guidelines to ensure that all key aspects of model development and risk of bias were comprehensively addressed and reported.

## 3. Results

### 3.1. Baseline characteristics of study participants

A total of 100 pregnant individuals with singleton gestations were included in this retrospective case-control study, consisting of 50 participants in the PE group and 50 in the control group. The baseline demographic and clinical characteristics of these participants are summarized in Table 1.

**Table 1:** Baseline characteristics of study participants.

Characteristic	Preeclampsia group (n = 50)	Control group (n = 50)	*P*-value
Age (yr), median (IQR)	32.5 (29–36)	31 (28–35)	.212
BMI (kg/m^2^), median (IQR)	24.8 (23.1–26.5)	24.1 (22.8–25.9)	.172
Smoking (n, %)	16 (32.0)	14 (28.0)	.315
Primiparity, n (%)	32 (64.0)	32 (64.0)	.362
Preeclampsia history, n (%)	6 (12.0)	2 (4.0)	.083
Systolic BP (mm Hg), median (IQR)	125 (107–132)	111 (101–114)	<.001
Diastolic BP (mm Hg) median (IQR)	78 (74–88)	69 (60–72)	<.001
Preterm birth (< 37 week; n, %)	14 (27.0)	5 (10.0)	<.001

BMI = body mass index, BP = blood pressure; mm Hg = millimeters of mercury, IQR = interquartile range.

The median maternal age in the PE group was 32.5 years (IQR: 29–36), which was comparable to the control group’s median age of 31 years (IQR: 28–35; *P* = .212). Similarly, the median body mass index did not differ significantly between the PE group (24.8 kg/m^2^; IQR: 23.1–26.5) and the control group (24.1 kg/m^2^; IQR: 22.8–25.9; *P* = .172). Smoking status was also similar between the groups, with 32.0% in the PE group and 28.0% in the control group reporting smoking (*P* = .315). The proportion of primiparous women was identical in both groups at 64.0% (*P* = .362). A history of PE was observed in 12.0% of the PE group compared to 4.0% of the control group, showing a nonsignificant trend (*P* = .083).

Early pregnancy SBP and DBP were significantly higher in the PE group. The median SBP was 125 mm Hg (IQR: 107–132) in the PE group versus 111 mm Hg (IQR: 101–114) in the control group (*P* < .001). Similarly, the median DBP was 78 mm Hg (IQR: 74–88) in the PE group compared to 69 mm Hg (IQR: 60–72) in the control group (*P* < .001). The incidence of preterm birth was significantly higher in the PE group, with 27.0% of participants experiencing preterm birth compared to only 10.0% in the control group (*P* < .001).

### 3.2. Early pregnancy indicator comparison

To identify potential early predictive biomarkers for PE, we collected and compared early pregnancy (prior to 12 weeks of gestation) indicators between the PE and control groups. These indicators encompassed a broad spectrum, including hematological, biochemical, inflammatory, angiogenic, and biophysical markers, as well as liver, kidney, and coagulation parameters. Detailed comparisons, including median (IQR) or mean ± standard deviation values and corresponding *P*-values, are provided in Table 2.

**Table 2:** Early pregnancy indicator comparison between preeclampsia and control groups.

Characteristic	Preeclampsia group (n = 50)	Control group (n = 50)	*P*-value
WBC-related parameters			
WBC count (10^9^/L)	9.5 (8.2–11.0)	7.5 (6.3–9.0)	.002
Neutrophil count (10^9^/L)	7.0 (5.8–8.5)	4.8 (3.9–6.0)	<.001
Lymphocyte count (10^9^/L)	1.5 (1.2–1.8)	2.0 (1.7–2.3)	.003
Monocyte count (10^9^/L)	0.62 (0.48–0.91)	0.49 (0.37–0.66)	.005
Eosinophil count (10^9^/L)	0.15 (0.10–0.20)	0.12 (0.08–0.18)	.156
Basophil count (10^9^/L)	0.03 (0.02–0.04)	0.03 (0.02–0.04)	.870
C-reactive protein (CRP; mg/L)	7.8 (5.5–10.2)	2.5 (1.8–3.5)	<.001
RBC-related parameters			
RBC count (10^12^/L)	4.0 (3.8–4.2)	4.1 (3.9–4.3)	.189
Hemoglobin (Hb; g/dL)	10.5 (9.8–11.2)	11.8 (11.0–12.5)	.001
Hematocrit (HCT; %)	32.0 (30.5–33.5)	35.5 (34.0–37.0)	.002
MCV (fL)	80.5 (78.0–83.0)	81.0 (79.0–84.0)	.450
MCH (pg)	27.5 (26.0–28.5)	28.0 (27.0–29.0)	.321
MCHC (g/dL)	33.0 (32.5–33.5)	33.2 (32.8–33.8)	.610
RDW-CV (%)	14.5 (13.8–15.2)	12.8 (12.0–13.5)	<.001
RDW-SD (fL)	48.0 (45.0–51.0)	44.0 (42.0–46.0)	.075
Derived calculated indices			
NLR	4.7 (3.8–5.5)	2.4 (2.0–2.8)	<.001
MLR	0.45 (0.38–0.52)	0.37 (0.21–0.34)	.071
PLR	180 (160–200)	177 (124–188)	.054
PC/MPV	15.0 (13.5–16.5)	15.2 (13.8–16.8)	.780
Angiogenic factors			
PlGF (pg/mL)	150 (120–180)	380 (320–450)	<.001
Biophysical markers			
Uterine artery PI	2.5 (2.0–3.0)	1.5 (1.2–1.8)	<.001
Liver function indicators			
ALT (U/L)	35 (28–42)	34 (22–45)	.073
AST (U/L)	30 (25–35)	22 (18–26)	.002
LDH (U/L)	200 (180–220)	190 (170–210)	.250
ALB (g/L)	38 (36–40)	39 (37–41)	.410
Kidney function indicators			
Cr (µmol/L)	65 (60–70)	55 (50–60)	.005
BUN (mmol/L)	4.5 (4.0–5.0)	4.2 (3.8–4.8)	.180
UA (µmol/L)	369.65 (311.15–454.61)	334.57 (299.84–371.42)	.001
Coagulation parameters			
FIB (g/L)	4.2 (3.8–4.6)	4.1 (3.7–4.5)	.550
d-dimer (ug/mL)	720.11 (418.6–957.4)	684.14 (314.5–862.4)	.002

ALB = albumin, ALT = alanine aminotransferase, AST = aspartate aminotransferase, BUN = blood urea nitrogen, Cr = creatinine, CRP = C-reactive protein, FIB = fibrinogen, Hb = hemoglobin, HCT = hematocrit, LDH = lactate dehydrogenase, MCH = mean corpuscular hemoglobin, MCHC = mean corpuscular hemoglobin concentration, MCV = mean corpuscular volume, MLR = monocyte-to-lymphocyte ratio, NLR = neutrophil-to-lymphocyte ratio, PC/MPV = platelet count to mean platelet volume ratio, PI = pulsatility index, PlGF = placental growth factor, PLR = platelet-to-lymphocyte ratio, RBC = red blood cell, RDW-CV = red cell distribution width-coefficient of variation, RDW-SD = red cell distribution width-standard deviation, UA = uric Acid, WBC = white blood cell.

WBC related parameters showed several significant differences. The PE group had significantly higher median WBC count (*P* = .002), neutrophil count (*P* < .001), monocyte count (*P* = .005), and CRP levels (*P* < .001) compared to the control group, suggesting a heightened systemic inflammatory state in early pregnancy among women who later developed PE. Conversely, lymphocyte count was significantly lower in the PE group (*P* = .003), while eosinophil and basophil counts did not show significant differences.

Among RBC related parameters, the PE group exhibited significantly lower median Hb (*P* = .001) and hematocrit (*P* = .002) values. RDW-CV was significantly higher in the PE group (*P* < .001), indicating greater red cell heterogeneity. Other RBC parameters such as RBC count, mean corpuscular volume, mean corpuscular hemoglobin, and mean corpuscular hemoglobin concentration did not show significant differences.

Derived calculated indices, reflecting systemic inflammation and immune status, were notably altered. The NLR (*P* < .001) was significantly higher in the PE group. While MLR, and PLR and the platelet count to mean platelet volume ratio did not show a statistically significant difference (*P* > .05).

Angiogenic factors revealed a crucial difference, with the PE group having significantly lower median PlGF levels (*P* < .001) compared to the control group. Similarly, the biophysical marker, UtAPI, was significantly higher in the PE group (*P* < .001), indicating increased uterine vascular resistance.

As for liver function indicators, the PE group demonstrated significantly higher median AST (*P* = .002) levels, suggesting early hepatic involvement. Lactate dehydrogenase and serum albumin did not show significant differences. Kidney function indicators also revealed higher median serum creatinine (Cr; *P* = .005) and UA (*P* = .001) in the PE group, although blood urea nitrogen was not significantly different.

In terms of coagulation parameters, the PE group exhibited significantly higher median d-dimer levels (*P* = .002), indicative of increased fibrin turnover, while fibrinogen levels were not significantly different.

### 3.3. Feature selection by LASSO and performance of individual selected indicators

To identify the most parsimonious set of relevant early pregnancy predictors of PE, we performed LASSO regression on all 30 collected theoretical indicators.

**Figure 1.**
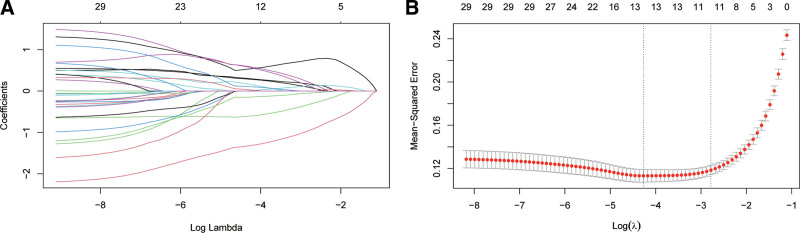
Feature selection using LASSO regression. (A) The regularization path of the coefficients for the predictors. (B) Cross-validation results for tuning parameter selection. LASSO = least absolute shrinkage and selection operator.

Through LASSO regression, a subset of 12 key early pregnancy indicators was identified as having nonzero coefficients, indicating their significant predictive power for PE (Fig. 1). These selected features included: PlGF, UtAPI, CRP, NLR, d-dimer, Hb, RDW-CV, AST, UA, early pregnancy SBP, early pregnancy DBP, and monocyte count.

**Figure 2.**
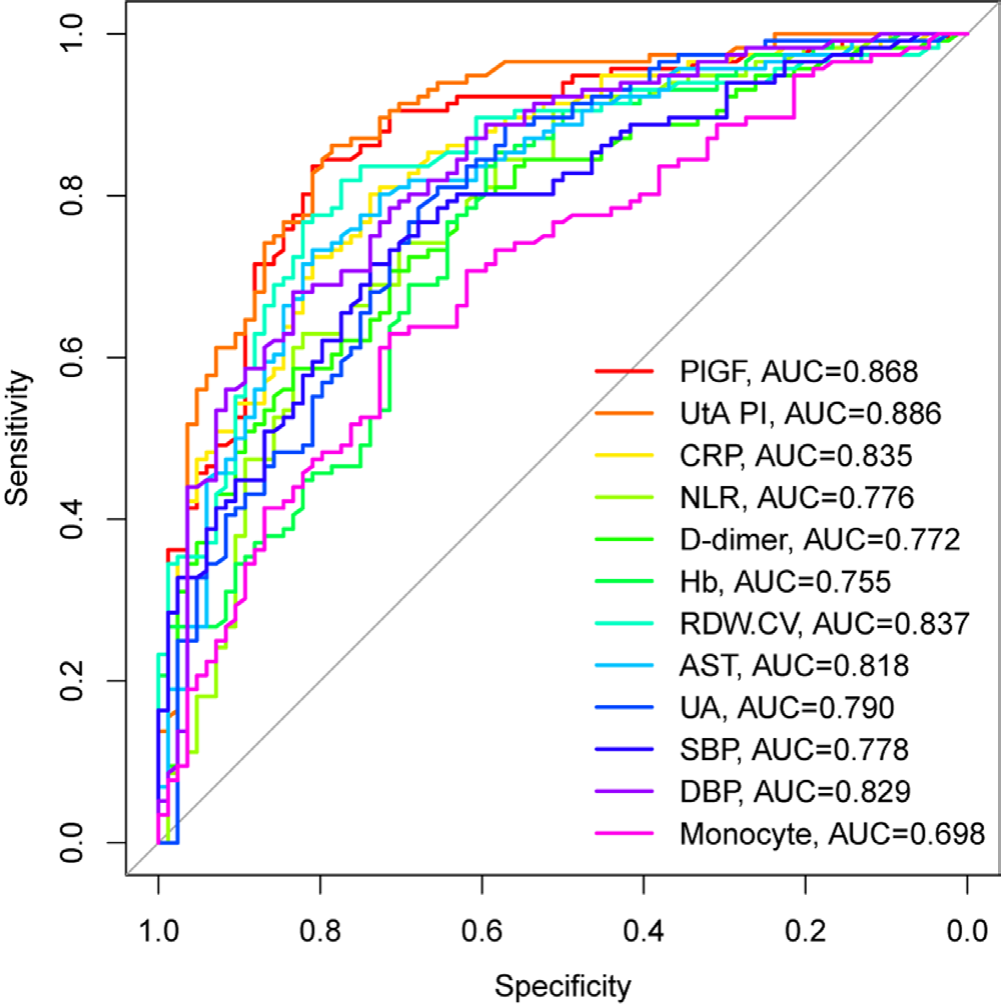
Receiver operating characteristic (ROC) curves for individual early pregnancy indicators in predicting preeclampsia. AUC = area under the curve, AST = aspartate aminotransferase, CRP = C-reactive protein, DBP = diastolic blood pressure, Hb = Hemoglobin, NLR = neutrophil-to-lymphocyte ratio, PlGF = placental growth factor, RDW-CV = red cell distribution width-coefficient of variation, SBP = systolic blood pressure, UA = uric acid, UtAPI = uterine artery pulsatility index.

We then conducted ROC curve analysis for each of these 12 selected individual indicators to evaluate their discriminative performance in predicting PE (Fig. 2). Among the individual markers, UtAPI demonstrated the highest discriminative ability (area under the curve [AUC] = 0.886), followed closely by PlGF (AUC = 0.868) and RDW-CV (AUC = 0.837). Other markers showing strong performance included CRP (AUC = 0.835), DBP (AUC = 0.829), and AST (AUC = 0.818). Further indicators such as UA (AUC = 0.790), SBP (AUC = 0.778), NLR (AUC = 0.776), d-dimer (AUC = 0.772), and Hb (AUC = 0.755) also demonstrated good individual predictive performance. Monocyte count showed the lowest AUC among the selected indicators (AUC = 0.698).

### 3.4. Performance comparison of ML models

Following the identification of the 12 key early PE indicators by LASSO, these selected features were then used to develop and compare the performance of 7 different ML models for PE prediction. The models investigated included RF, SVM, GLM (referring to logistic regression here), GBM, KNN, NNET, and DT. Each model was trained and optimized through *k*-fold repeated cross-validation (5-fold, repeated 5 times) on the training dataset to fine-tune hyperparameters and ensure robust internal validation, minimizing overfitting.

**Figure 3.**
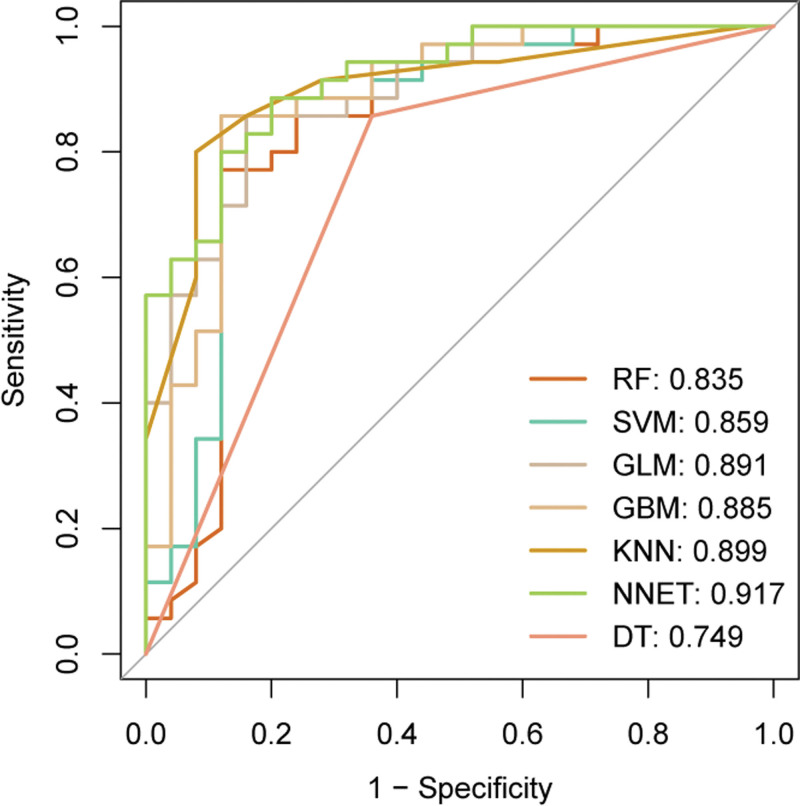
Receiver operating characteristic (ROC) curves of different machine learning models for preeclampsia prediction. DT = decision tree, GBM = gradient boosting machine, GLM = generalized linear model, KNN = K-nearest neighbors, NNET = neural network, RF = random forest, SVM = support vector machine.

The predictive performance of all 7 developed ML models was rigorously evaluated based on their ability to predict PE on the unseen test set. Figure 3 illustrates the ROC curves for each model, with their discriminative ability quantified by the AUC. The NNET model demonstrated the highest AUC (0.917), indicating its superior predictive distinction compared to the other models. Following NNET, the performance hierarchy was KNN (AUC = 0.899), GLM (AUC = 0.891), GBM (AUC = 0.885), SVM (AUC = 0.859), RF (AUC = 0.835), and DT (AUC = 0.749).

**Figure 4.**
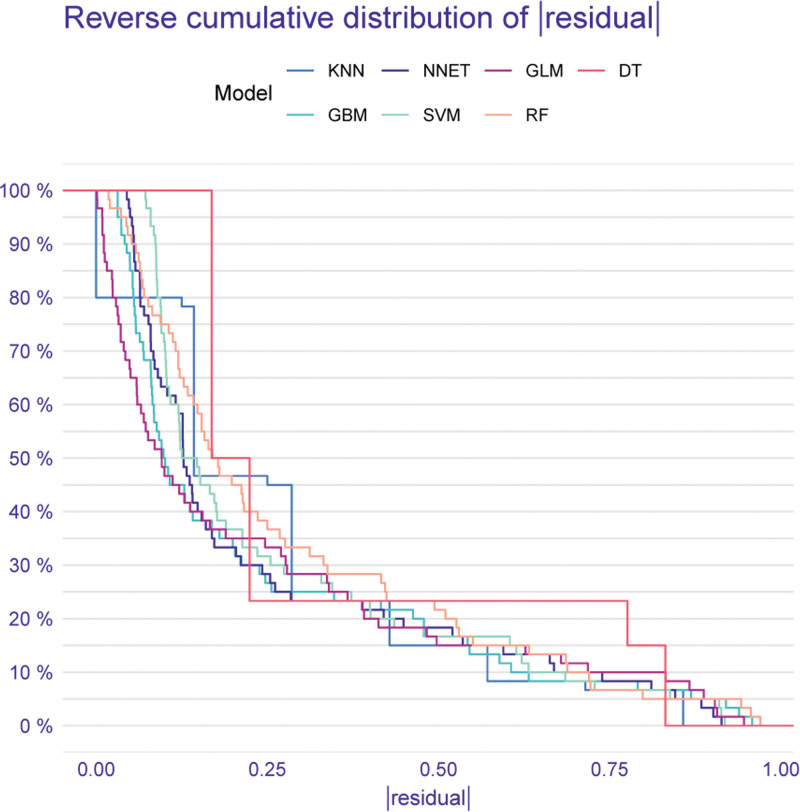
Reverse cumulative distribution of residuals for different machine learning models. DT = decision tree, GBM = gradient boosting machine, GLM = generalized linear model, KNN = K-nearest neighbors, NNET = neural network, RF = random forest, SVM = support vector machine.

Further assessment of model performance was conducted using the reverse cumulative distribution of residuals, as shown in Figure 4. This plot visually represents the distribution of prediction errors, where curves positioned further towards the top-left indicate a higher proportion of predictions with smaller residuals (i.e., more accurate predictions). Consistent with the AUC results, the NNET model exhibited superior performance in this analysis, showing a greater percentage of residuals closer to zero.

### 3.5. Feature importance and interpretability of the best model

To visually and quantitatively present the contribution of the selected variables, we utilized the SHAP package to analyze the best-performing NNET predictive model. SHAP values illustrate each feature’s positive and negative impact on the model’s output for a given sample.

**Figure 5.**
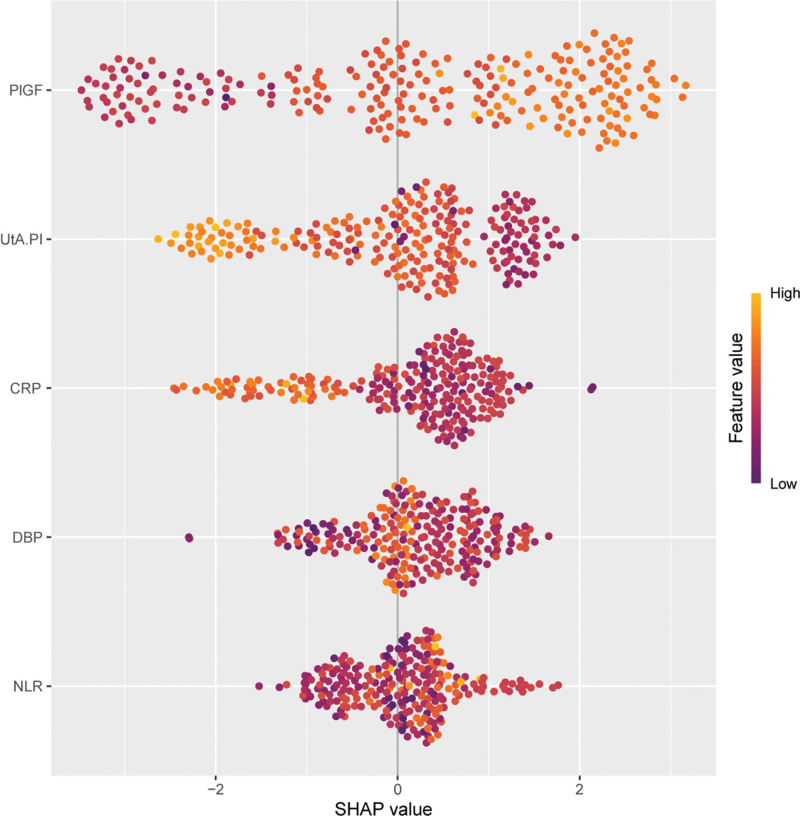
SHAP (SHapley Additive exPlanations) summary plot illustrating feature contributions to the neural network (NNET) model’s predictions. CRP = C-reactive protein, DBP = diastolic blood pressure, NLR = neutrophil-to-lymphocyte ratio, PlGF = placental growth factor, UtAPI = uterine artery pulsatility index, .

Figure 5 displays the mean absolute SHAP values for the different features, quantifying their average magnitude of impact on the NNET model’s prediction. PlGF, UtAPI, CRP, early pregnancy DBP, and NLR emerged as the most significant drivers of the prediction within the NNET model.

### 3.6. Performance of the NNET model on the external validation cohort

**Figure 6.**
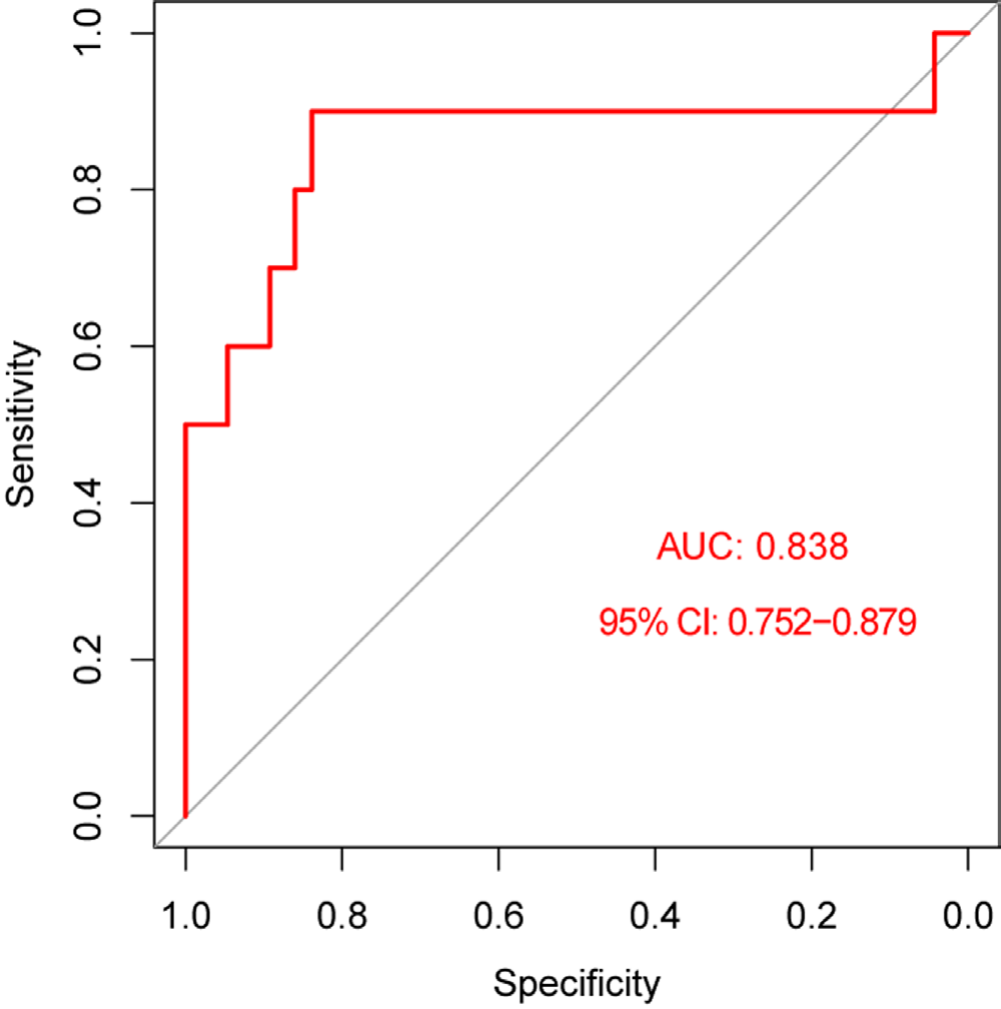
Receiver operating characteristic (ROC) curve of the neural network (NNET) model on the external validation cohort. AUC = area under the curve.

The selected NNET predictive model, trained on the development cohort, was applied to the independent external validation cohort. The model demonstrated robust performance on this new dataset, achieving an AUC the ROC of 0.838 (95% CI: 0.752–0.879; Fig. 6), whose performance was comparable to its performance on the internal test set (AUC = 0.917).

## 4. Discussion

PE remains a significant cause of maternal and perinatal morbidity and mortality, particularly in low- and middle-income countries, with an increasing global incidence due to rising maternal age and comorbidities such as obesity and diabetes.^[17,18]^ Early prediction of PE is crucial for the timely implementation of preventive measures, which could significantly alter its clinical course and improve outcomes for both mother and child.^[19,20]^ In our study, we developed a predictive model using a broad range of maternal, biophysical, biochemical, and hematological indicators from the 1st trimester to predict the risk of PE. The model’s predictive performance, evaluated using ML techniques including LASSO regression for feature selection and subsequent training of 7 different models (with NNET identified as the best performer), demonstrated superior accuracy, particularly when combined with clinical features and biomarkers that reflect systemic inflammation and endothelial dysfunction.

Our findings corroborate existing evidence linking systemic inflammation to PE. Elevated NLR, monocyte counts, and CRP levels in early pregnancy have been repeatedly identified as markers of increased inflammatory responses, which are believed to play a crucial role in the pathophysiology of PE.^[21–23]^ Several studies have highlighted the role of inflammation in endothelial dysfunction and impaired vascular remodeling, which are central to PE development.^[10,24]^ Similarly, elevated CRP levels in PE patients, as observed in our study, have been previously associated with poor pregnancy outcomes and have been suggested as potential biomarkers for PE prediction.^[25]^

In addition to inflammatory markers, our results also demonstrate significant differences in angiogenic factors, PlGF, which was found to be significantly lower in the PE group. This finding aligns with numerous studies that have shown a decrease in PlGF as a hallmark of PE and its association with poor placental perfusion and subsequent hypertension.^[26,27]^ Reduced PlGF levels reflect impaired trophoblastic invasion and inadequate remodeling of the spiral arteries, which are characteristic of the pathophysiology of PE.^[28,29]^ Several studies have further demonstrated that low PlGF concentrations, especially when combined with elevated levels of soluble fms-like tyrosine kinase-1 (sFlt-1), significantly improve the prediction of PE, particularly in early-onset cases.^[30,31]^ The sFlt-1/PlGF ratio has been widely adopted as a robust biomarker for diagnosing and stratifying PE severity.^[32,33]^

Moreover, PlGF is also gaining attention for its utility in longitudinal monitoring of high-risk pregnancies. For instance, a prospective study by Stefan Verlohren et al demonstrated that a low PlGF or high sFlt-1/PlGF ratio could predict the development of PE within 1 to 4 weeks in women presenting with clinical symptoms.^[34]^ In the context of 1st-trimester screening, combining PlGF with other parameters such as UtAPI, maternal serum pregnancy-associated plasma protein-A levels, and maternal clinical characteristics has yielded predictive models with high sensitivity and specificity.^[35,36]^ These multimarker approaches underscore the importance of PlGF not only as a standalone marker but also as part of integrated risk assessment tools.

Our ML approach, particularly the use of LASSO regression, enabled the identification of key predictive features, improving the sensitivity and accuracy of PE prediction models. The integration of multiple predictors, such as PlGF, UtAPI, CRP, and NLR, resulted in a robust model with high discriminative performance (AUC = 0.917). The NNET model, in particular, outperformed other ML algorithms, demonstrating superior predictive accuracy compared to traditional logistic regression and DT models. The use of SHAP values in our analysis allowed for better interpretability of the model, revealing that PlGF, UtAPI, CRP, and NLR were the most influential features driving the prediction. This aligns with prior studies that have also identified these markers as central to PE pathophysiology and prediction.^[37–39]^

To provide a more comprehensive context for our findings, it is essential to compare our results with other ML-based prediction models for PE, many of which have been published in recent years.^[40]^ A recent systematic review by Ranjbar et al highlighted the growing use of ML models for PE prediction, with reported AUCs ranging from 0.64 to 0.96, depending on the study design and input features.^[41]^ Our NNET model’s high AUC of 0.917 for our internal cohort is highly competitive with, and in some cases, superior to models developed in other studies. For instance, a study by Jhee et al developed a ML model for late-onset PE using a large cohort of over 11,000 pregnant women, achieving a best-model C-statistic (equivalent to AUC) of 0.924.^[42]^ Similarly, a retrospective cohort study in China by Liu et al^[43]^ used 5 ML algorithms and found their RF model to have the highest AUC of 0.86. Marić et al,^[40]^ using data from over 16,000 births, developed an elastic net model with an AUC of 0.89 for early-onset PE. Li et al developed models using electronic health records and reported a best-model AUC of 0.955 for an XGBoost model.^[44]^ The high predictive performance of our model likely stems from the carefully selected combination of biophysical (UtAPI), biochemical (PlGF), and inflammatory (CRP, NLR) markers, which collectively capture multiple facets of PE pathophysiology. Our NNET model’s superiority can be attributed to its ability to capture complex, nonlinear relationships between these diverse predictors. Unlike traditional linear models (such as the GLM/logistic regression model in our study, which had an AUC of 0.891), NNETs excel at identifying intricate patterns in high-dimensional data, which is characteristic of the multifactorial nature of PE. These comparisons suggest that the specific feature combination we identified via LASSO regression, coupled with the pattern recognition capabilities of the NNET algorithm, offers a particularly robust approach.

Despite its strengths, this study has several limitations. Firstly, as a single-center retrospective cohort study, our sample size of 100 participants is relatively small compared to large-scale studies, which may limit the generalizability of our findings to more diverse populations. While we employed an independent external validation cohort, further multi-center prospective studies are needed to confirm these results on a broader scale. Secondly, although we included a broad range of indicators, our model did not account for all potential influencing factors. This includes the lack of inclusion of pregnancy-associated plasma protein-A, which is a key marker in 1st-trimester screening for PE in many established models. Future research should aim to incorporate this and other factors, such as specific genetic predispositions, nutritional status, and detailed lifestyle factors, to enhance model accuracy. Future research should aim to incorporate these factors to enhance model accuracy. Lastly, while ML models offer high predictive power, their “black-box” nature can sometimes hinder direct clinical interpretation. Although we addressed this using SHAP values, further work on developing more inherently interpretable models or more intuitive visualization tools for complex ML models in clinical settings would be beneficial.

## 5. Conclusion

In conclusion, we successfully developed and validated a comprehensive predictive model for PE using early pregnancy indicators and ML techniques. Our NNET model, utilizing 12 LASSO-selected features, demonstrated excellent discriminative ability and robust performance on an independent external validation cohort. Key predictors including PlGF, UtAPI, CRP, DBP, and NLR were identified as the most influential factors driving the model’s predictions. This system holds promise for early identification of high-risk pregnancies, enabling timely interventions and ultimately improving maternal and fetal outcomes, though further large-scale prospective validation is warranted.

## Author contributions

**Conceptualization:** Haixia Liang, Xuejing Zhao, Ying Zhang, Yujie Wu, Han Wu, Ying He.

**Formal analysis:** Zehui Zhang.

**Funding acquisition:** Zehui Zhang.

**Investigation:** Xuejing Zhao, Han Wu.

**Methodology:** Xuejing Zhao, Ying Zhang, Yujie Wu.

**Resources:** Ying Zhang, Yujie Wu.

**Software:** Han Wu, Zehui Zhang, Ying He.

**Supervision:** Xuejing Zhao, Ying Zhang, Yujie Wu, Han Wu, Zehui Zhang, Ying He.

**Validation:** Xuejing Zhao, Yujie Wu, Han Wu.

**Visualization:** Haixia Liang.

**Writing – original draft:** Haixia Liang.

**Writing – review & editing:** Ying Zhang, Han Wu, Zehui Zhang, Ying He.
